# Facilitators of and Barriers to Successful Implementation of the One Key Question^®^ Pregnancy Intention Screening Tool

**DOI:** 10.1089/whr.2021.0100

**Published:** 2022-03-08

**Authors:** Meron Ferketa, Kellie Schueler, Bonnie Song, Francesca Carlock, Debra B. Stulberg, Emily White VanGompel

**Affiliations:** ^1^ Pritzker School of Medicine, University of Chicago, Chicago, Illinois, USA.; ^2^ NorthShore University HealthSystem (NSUHS) Research Institute, Evanston, Illinois, USA.; ^3^ Department of Family Medicine, University of Chicago, Chicago, Illinois, USA.

**Keywords:** preconception counseling, contraceptive counseling, pregnancy intentions, primary care, implementation science

## Abstract

**Background:**

One Key Question^®^ (OKQ) is a tool that embeds a patient-centered screening into routine visits with the goal of making pregnancy intention screening universal, but widespread implementation has not yet been adopted. We aimed to explore the barriers and facilitators of OKQ implementation to better understand how to best implement the tool across different settings.

**Methods:**

We invited staff and clinicians from one obstetrics and gynecology clinic and one family medicine clinic, which previously implemented OKQ, to complete surveys and qualitative interviews about their experiences with the tool. The interview guide and thematic analysis of the interview transcripts were informed by the Consolidated Framework for Implementation Research (CFIR).

**Main Findings:**

Major facilitators of OKQ implementation are the simplicity of the tool, engagement of clinic leadership, and compatibility between the perceived goals of the tool and those of practice staff and clinicians. Although participants indicated that OKQ had a minimal impact on clinic workflow during its implementation, preimplementation time concerns were a major barrier to implementation in both clinics. Barriers seen in the family medicine practice included OKQ distracting from the visit agenda, and concerns about the OKQ gold standard protocol of screening each patient at every visit. Participants even suggested asking OKQ only during annual check-up appointments.

**Conclusions:**

The perceived alignment between the tool's goals and those of clinic stakeholders was an important facilitator of OKQ implementation success. However, characteristics of the clinic setting, such as competing medical priorities and time constraints, influenced initial attitudes toward the feasibility of the intervention. Clinical Trial Registration Number: NCT03947788

## Introduction

Maternal morbidity and mortality continue to rise in the United States, in part, due to health problems that exist before a person becomes pregnant.^1^ To optimize health outcomes for birthing people and their babies, the American College of Obstetrics and Gynecology (ACOG) and the Centers for Disease Control and Prevention (CDC) recommend that primary care clinicians routinely assess patients' reproductive health and provide contraceptive and/or preconception counseling accordingly.^1–3^ Actual practice remains far from this goal, however, with research from a nationally representative sample of ambulatory visits finding that nonpregnant, reproductive-aged women received family planning services in only 14% of visits from 2009 to 2010.^3^

Several provider- and systems-level barriers may contribute to low rates of reproductive health counseling in primary care settings.^4–6^ Provider-level barriers may include lack of knowledge, training, or comfort in reproductive health counseling, reliance on patients to initiate discussions, and assumptions about patient pregnancy risk.^3–8^ Systems-level barriers may include lack of time during office visits, and other competing medical issues.^4–8^ One potential solution to address these barriers is incorporating pregnancy intention screening as a routine part of clinical care, but evidence is lacking as to whether and how to best implement this.

One Key Question^®^ (OKQ) is a pregnancy intention screening tool used by providers to ascertain a patient's preferences surrounding pregnancy and provide reproductive counseling accordingly.^7,9^ The tool prompts patients to answer one question, “do you wish to become pregnant in the next year?,” and providers are trained to provide counseling based on patient responses of “yes,” “no,” “unsure,” or “ok either way.”^7,9^

Patient responses to the OKQ screening correlates with a validated 14-question measure of a patient's desire to avoid pregnancy,^10^ and due to its short length, it may be a practical tool for routine clinical use.^7^ One community health center that incorporated OKQ into its electronic medical record saw increases in contraceptive counseling and decreases in patient satisfaction.^11^

A recent pre- versus postintervention assessment of OKQ in a primary care and general obstetrics and gynecology (OB/GYN) practice showed increased patient satisfaction after implementation of OKQ, while satisfaction decreased in control practices over the same time period; but in these practices, OKQ had no significant effect on reproductive counseling.^12^ These conflicting results suggest that further in-depth understanding of how best to implement the screening tool is needed.

Research has shown that intervention effectiveness is influenced by certain setting characteristics, and the implementation strategy used.^13–15^ The Consolidated Framework for Implementation Research (CFIR) provides a framework to identify factors that may affect an intervention's implementation and effectiveness in different settings, and enable improved effectiveness and replication.^13^

Following the efficacy assessment of OKQ conducted from 2017 to 2019 referenced above,^12^ we undertook to evaluate the actual implementation process to inform and optimize future use. Using the CFIR framework, a comprehensive analysis identified individual- and systems-level barriers and facilitators experienced by clinicians and staff during implementation of OKQ.

## Materials and Methods

### Setting and participants

We invited staff and clinicians from two clinics to be surveyed and interviewed about their experience implementing OKQ during the pilot study. Both clinics, one family medicine and one OB/GYN, were associated with a large academically affiliated, nonprofit community hospital network located in the northern suburbs of Chicago, Illinois. The clinics differed in structure (the family medicine clinic included resident physicians, the OB/GYN clinic did not), and in size (the family medicine clinic had a larger staff size). We invited physicians, nurses, medical assistants, and administrators from each clinic to complete an anonymous survey about their experiences with OKQ.

We later recruited staff and clinicians by email to participate in qualitative interviews. We continued recruitment until saturation was achieved with adequate representation from both clinics and all clinic roles. Interviews were semistructured and lasted 30–45 minutes either by phone or in-person with a member of the research staff. All interviews were recorded and transcribed. Interview participants received a $50 dollar gift card in appreciation for their time. Informed consent was obtained from all participants, and our Institutional Review Board deemed the study exempt (approved December 20, 2019).

### Survey analysis

We designed a postimplementation survey to capture implementation concepts of feasibility, perceived appropriateness, and acceptability.^16^ Likert-style items asked participants' opinion on the usefulness of OKQ as a pregnancy intention screening tool and its impact on clinic workflow. Items were scored from 0, strongly disagree, to 4, strongly agree. Means were calculated for both clinics and between-group comparisons of means analyzed using *t*-tests.

One item on the survey asked physicians if they agreed or disagreed with the statement, “I needed to recommend a follow-up visit for reproductive health care.” Those who agreed or strongly agreed with this statement were asked to circle the reasons for scheduling the follow-up visit: time, patient preference, too many other items on the agenda, and/or care initiated but needs follow-up. Responses from the two clinics were compared using chi-squared tests.

Another item asked participants to write in their recommendations for changing the OKQ screening tool. Responses were analyzed for common themes.

### Interview analysis

#### Interview guide

We developed the interview guide to be informed by the CFIR framework, creating questions to address specific CFIR constructs. CFIR includes five domains: intervention characteristics, inner setting (features of the implementing organization), outer setting characteristics (features of the external environment), individual characteristics, and implementation processes.^13^ Each domain includes constructs that facilitate or impede intervention implementation. We asked participants about their attitude toward OKQ, its benefits and drawbacks as a pregnancy intention screening tool, issues with time or integration into clinic workflow, their thoughts on the OKQ training session, and other topics.

#### Codebook development and analysis

The preliminary codebook was developed by research team members, guided by the CFIR framework. Two coders independently coded the first three transcripts and each created a code list based on salient concepts. These initial lists were consolidated through team discussion with the senior author. The first six transcripts were then coded separately by the two initial coders using this consolidated codebook. All discrepancies were resolved through discussion among the coding team and the codebook was refined.

Using this finalized codebook, the remaining 17 transcripts were then divided and coded individually by one coder. All transcripts were coded using Dedoose, a qualitative data analysis software (Sociocultural Research Consultants, Los Angeles, CA).

To analyze the data, we reviewed and summarized the excerpts related to each frequently used code. From these code summaries, we integrated emergent themes into an explanatory narrative to determine important facilitators or barriers in the implementation of One Key Question.

## Results

### Survey results

From the participating clinics, 42 out of 54 staff/clinicians completed the survey for a response rate of 78%. Twenty-seven respondents (64%) worked at the family medicine clinic while 15 (36%) worked at the OB/GYN clinic. In terms of clinical roles, 20 (49%) physicians, 16 (39%) clinical support staff, and 5 (12%) nonclinical support staff completed the survey.

#### Appropriateness

Overall, a majority of respondents from both clinics felt that OKQ addressed an important clinical need (95% agreed or strongly agreed, Table 1).

**Table 1.** Responses of Family Medicine Versus Obstetrics and Gynecology Staff and Clinicians to the Following Statements

Statement	Practice type		
	Total, ***N*** (%)	Family medicine, ***n*** (%)	OB/GYN, ***n*** (%)
OKQ addressed an important clinical need (% who agree or strongly agree)	40 (95)	26 (96)	14 (93)
Patients appreciated being asked at the visit (% who agree or strongly agree)	37 (88)	23 (85)	14 (93)
I would like to see this tried with a larger sample size (% who agree or strongly agree)	36 (86)	25 (93)	11 (73)
The intervention excessively slowed check-in time (% who disagree or strongly disagree)	38 (90)	25 (93)	13 (87)
The intervention excessively slowed room turnover (% who disagree or strongly disagree)	39 (93)	25 (93)	14 (93)
The tool excessively lengthened visit length (% of physicians who disagree or strongly disagree)	18 (90)	14 (88)	4 (100)

No significant differences between responses of Family Medicine and OB/GYN were found.

OB/GYN, obstetrics and gynecology; OKQ, One Key Question^®^.

#### Feasibility

Most respondents also felt that OKQ did not excessively slow down room turnover time (93% disagreed or strongly disagreed when asked if OKQ excessively slowed room turnover, Table 1) or lengthen the check in process (90% disagreed or strongly disagreed when asked if OKQ excessively lengthened check in time, Table 1). Of the physicians surveyed from both clinics, 90% disagreed or strongly disagreed when asked if OKQ excessively lengthened the clinic visit (Table 1).

**FIG. 1.**
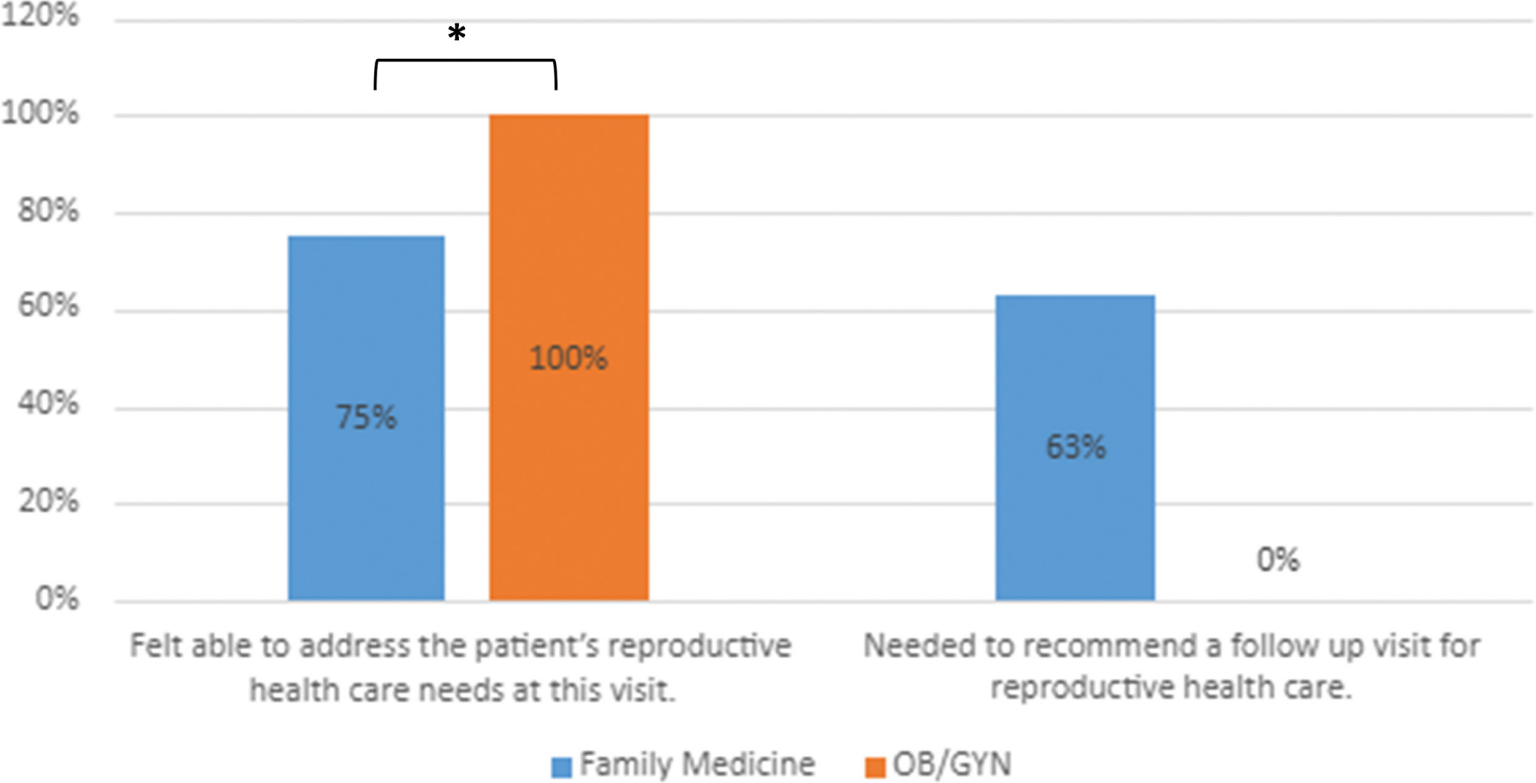
Percentage of Family Medicine versus OB/GYN clinicians who agreed with the following statements. OB/GYN, obstetrics and gynecology.*Student's *t*-test; *p* < .01.

Despite most physicians feeling that they were able to address patients' reproductive needs during the current health care visit (80% agreed or strongly agreed), there was a significant difference between clinics with OB/GYN clinicians feeling that they were more able to address the topic during the visit versus family medicine clinicians (Family Medicine Mean = 2.9 vs. OB/GYN Mean = 4.0, *p* = 0.01; Fig. 1).

Although not a statistically significant difference, only family medicine physicians indicated that they needed to recommend follow-up visits for reproductive care versus no OB/GYN physicians (Family Medicine Mean = 2.6 versus OB/GYN Mean = 1.8, *p* = 0.06; Fig. 1). These physicians cited time concerns (90%), too many other items on the agenda (70%), and follow-up needed for initiated care (30%) as reasons for recommending follow-up visits with patients.

#### Acceptability

The majority of respondents from both clinics felt that patients appreciated being asked about their reproductive needs through OKQ (88% agreed or strongly agreed, Table 1). In addition, most respondents agreed or strongly agreed that they would like to see OKQ tried out on a larger sample size (86%, Table 1).

Notably, 21% of providers had changes that they would recommend to the OKQ screening tool. The most common recommended change was to give the screener only at well adult/preventive care visits.

### Interview results

A total of 23 clinicians and staff completed interviews (Table 2 for demographic information). Participants described both facilitators and barriers experienced during OKQ implementation (discussed below and summarized in Table 3).

**Table 2.** Demographic Characteristics (***n*, %) of Interview Participants (*n* = 23)**

Characteristic	Total, ***N*** (%)	Family medicine clinic, ***n*** (%)	OB/GYN clinic, ***n*** (%)
Total	23	15	8
Role			
Attending	10 (43)	6 (40)	4 (50)
Resident	5 (22)	5 (33)	0 (0)^a^
MA/NP	7 (30)	4 (27)	3 (38)
Administrator	1 (4)	0 (0)	1 (13)
Gender			
Male	3 (13)	2 (13)	1 (13)
Female	20 (87)	13 (87)	7 (88)
Total years in practice, years			
≤5	8 (35)	8 (53)	0 (0)
>5	15 (65)	7 (47)	8 (100)
Length of employment at study clinic, years			
≤5	12 (52)	10 (67)	2 (25)
>5	11 (48)	5 (33)	6 (75)
Attended OKQ training	16 (70)	8 (53)	8 (100)

^a^ OB/GYN clinic did not contain any resident physicians.

**Table 3.** Summary of Qualitative Data

Domain	Construct	Subconstruct	Theme	Additional illustrative quotes
Facilitators				
Intervention characteristics	Complexity: Perceived difficulty of the intervention, reflected by duration, scope, radicalness, disruptiveness, centrality, and intricacy and number of steps required to implement.		Simplicity of OKQ Minimal Workflow Impact	“So I think just the fact that it's just one simple question. That in itself is just the ease.”—Family Medicine Resident “I think in retrospect, not very largely… I think for the most part it happened while I was seeing a different patient so it wasn't as big of an issue.”—Family Medicine Resident
Inner setting	Readiness for Implementation: Tangible and immediate indicators of organizational commitment to its decision to implement an intervention	Leadership engagement: Commitment, involvement, and accountability of leaders and managers with the implementation.	Practice Leadership	
Process	Engaging: Attracting and involving appropriate individuals in the implementation and use of the intervention through a combined strategy of social marketing, education, role modeling, training, and other similar activities.	Champions: “Individuals who dedicate themselves to supporting, marketing, and ‘driving through’ an [implementation]” [101] (p. 182), overcoming indifference or resistance that the intervention may provoke in an organization.	Champions (and other supporters of OKQ)	“Just reminding people to do it and modeling the behavior… And then when people expressed frustration, to kind of walk it back and say this is the purpose of what we're doing and remind everybody of that intent.”—Family Medicine Resident
Inner setting	Implementation climate: The absorptive capacity for change, shared receptivity of involved individuals to an intervention, and the extent to which use of that intervention will be rewarded, supported, and expected within their organization.	Compatibility: The degree of tangible fit between meaning and values attached to the intervention by involved individuals, how those align with individuals' own norms, values, and perceived risks and needs, and how the intervention fits with existing workflows and systems.	Benefits of OKQ as Pregnancy Intention Screening Tool	“…you can capture those people who are… outliers of the reproductive age range… And you're assuming that those women don't want to conceive, some of them might want it as well.”—OB/GYN Attending Physician “…With One Key Question everybody got it…[Before OKQ] I think it was more discussed at well visits, but not necessarily at these other hospital follow-up or acute visits.”—Family Medicine Attending
Barriers				
Inner setting	Implementation climate: The absorptive capacity for change, shared receptivity of involved individuals to an intervention, and the extent to which use of that intervention will be rewarded, supported, and expected within their organization.	Compatibility: The degree of tangible fit between meaning and values attached to the intervention by involved individuals, how those align with individuals' own norms, values, and perceived risks and needs, and how the intervention fits with existing workflows and systems.	Time Concerns OKQ Distracting from Agenda Feasibility of Gold Standard	

Definitions of the Consolidated Framework for Implementation Research constructs and subconstructs from Damschroder et al.^13^ Additional illustrative quotes (not already mentioned in the text) included. CFIR.

#### Facilitators

##### Complexity

One important facilitator of OKQ implementation was the simplicity of the tool itself. Several participants described OKQ as a simple easy to use tool. In addition, most participants (including MAs, residents, and attendings from both clinics) stated that OKQ implementation had minimal impact on clinic workflow.

##### Readiness for implementation: leadership engagement

The engagement of practice leadership was another facilitator of OKQ implementation. Practice leadership promoted consistent use of the tool in multiple ways. First, leadership displayed their support for the intervention by involving staff and clinicians in implementation planning.

In this way, leadership attempted to get staff and clinicians involved and invested in the successful implementation of OKQ to motivate them to use the tool in their everyday practice. In addition, several participants stated that practice leadership solicited feedback about the intervention and made themselves available to address questions or concerns, particularly during meetings and team huddles.

##### Engaging: champions

CFIR defines champions as individuals who support an intervention and work to overcome organizational indifference or resistance to an intervention during implementation.^13^ Participants noted multiple ways that supporters of OKQ (including champions, attending physicians, and practice leadership) promoted its use. For example, precepting physicians at the family medicine practice would remind residents to ask OKQ. In addition, supporters would counter frustration about OKQ by reminding individuals of the reason behind the practice's decision to participate in the study. A family medicine resident stated:

“[Refocusing on] the point of this study…this is what our initial data shows that we aren't doing these things that everybody thought that they were doing really well at. And so kind of reframing it and putting it into perspective.”

##### Implementation climate: compatibility

Another major facilitator of implementation was the perceived benefits of OKQ and how these benefits aligned with the goals and values of key stakeholders. Such perceived benefits included an increase in pregnancy intention screening, an increase in preconception counseling, and opportunity for earlier clinician intervention.

During implementation of the intervention, both clinics attempted to follow the OKQ gold standard by developing workflows, in which all women of reproductive age (aged 18–50 years) were asked OKQ at each clinic visit. Many participants from both clinics perceived that OKQ increased the frequency with which patients were screened for pregnancy intention by expanding the patient population normally screened and encouraging screening across all visit types (including nonreproductive problem visits).

Moreover, participants perceived an increase, as well as a reframing, in their preconception counseling after implementation of OKQ. Many physicians from the family medicine clinic stated that before OKQ implementation, they did not address preconception counseling in their practice. One explanation for this perceived increase in preconception counseling is that OKQ reframed the way in which clinicians were asking patients about their reproductive health needs. One family medicine physician stated:

“[Before OKQ] I did a lot more of the contraception [counseling]. Like, ‘Have you thought about what you want to do for contraception,’ which was a different question than saying, ‘Hey, do you plan to get pregnant in the next year?’ Because you're addressing both things in that one question.”

Instead of focusing on contraceptive access and use, physicians were required to ask more generally about reproductive goals through the language of OKQ.

Also, participants from both the family medicine clinic and OB/GYN clinic perceived that OKQ was useful in initiating discussions about patients' reproductive goals and provided clinicians with earlier opportunities to provide counseling or options that support those goals. One OB/GYN physician stated:

“So I think [OKQ] opens up a conversation and ultimately that's more efficient than not, because instead of having the pregnancy scare phone call, the plan B phone in … or the management of the whole termination.”

Instead of managing an unplanned pregnancy, the clinician is able to discuss reproductive goals with the patient early on and provide advice that allows the patient to optimize their behaviors to support those goals. One family medicine physician states:

“The purpose of the One Key Question screening tool, as I see it, is to assess pregnancy planning and intendedness for women, undifferentiated women, of childbearing age… but also to empower women to know that they can make a choice and then adopt behaviors to support that choice in pregnancy planning.”

#### Barriers

##### Implementation climate: compatibility

Major barriers to OKQ implementation focused on concerns about how OKQ would integrate into clinic workflow. First, participants from both clinics mentioned having preimplementation concerns that OKQ would increase visit lengths or add to the workload of clinicians and staff. One OB/GYN physician stated:

“We were worried about the time consumption of it … all those things are very, very important when trying to implement any new program, is how is it adding on to the workload, and will it take away from something else.”

These preimplementation concerns contrast with participants' actual experiences with the tool during implementation. Participants across clinical roles (MAs, residents, attendings) and between clinic types (family medicine and OB/GYN) perceived OKQ as having a minimal impact on clinic workflow during its actual implementation (Tables 1 and 3).

However, there were differences between the family medicine and OB/GYN clinics. For example, implementation barriers such as OKQ distracting from the visit agenda and concerns about the practicality of the OKQ gold standard protocol were mentioned only by family medicine participants. Several family medicine clinicians recalled how OKQ interfered with or distracted from patients' visit agenda. One family medicine physician stated:

“… you would walk into the room and flip over the sheet and take a look at it in front of the patient and wouldn't really get time before you see the patient to plan for how will this fit into our visit agenda. Compared to all the other stuff that I planned like, where is this going to take priority depending on not just what I have planned and how urgent that is but how urgent is the answer to this question. Totally derailed the visit.”

Participants felt that this distraction or derailment was an issue particularly during problem-focused visits for nonreproductive health topics. One family medicine physician stated that in visits where the patient had a particularly significant or urgent agenda item, she would not address OKQ at all.

Furthermore, a few family medicine participants thought that the OKQ gold standard protocol of asking reproductive aged patients about pregnancy intention at every visit was inefficient and unnecessary. Instead, these participants suggested asking OKQ only at annual check-up appointments. One medical assistant stated:

“… I felt that it should be more focused on annual physicals… Because if they're coming in for a cough and they were given that question or like, ‘Oh, do you plan on getting pregnant within the next year?’ ‘Well that's not what I came in here for, I came in here for a cough.’”

## Discussion

This implementation study supports the acceptability, feasibility, and appropriateness of use of OKQ as a screening tool. Major factors that facilitated OKQ implementation included simplicity of the tool, leadership engagement, champions, and compatibility between the perceived goals of the tool and those of key practice stakeholders. Major barriers included preimplementation time concerns, issues with OKQ distracting from the visit agenda, and concerns about the OKQ gold standard protocol.

A key facilitator of the use of this tool was the ease with which participants saw it as compatible with their values and professional role. This study adds clinician perspective to similar findings among people of reproductive age that more inclusive conversations around reproductive goals are more acceptable than those focused solely on contraception.^17^ OKQ allows for patients to express interest, uncertainty, or ambivalence about a future pregnancy and provide opportunity for the physician to provide more patient-centered counseling.^18^ These tool characteristics may explain why the efficacy study of OKQ demonstrated increased patient satisfaction.^12^ Leveraging this dual beneficial characteristic may be a powerful means to facilitate uptake among more resistant clinics.

The results of this study reflect previous research on systems-level barriers, such as lack of time and competing medical priorities, encountered in incorporating reproductive health counseling in primary care.^3–8^ These concerns were especially prominent in the family medicine practice, in which staff and clinicians were not convinced of the OKQ gold standard protocol and suggested screening for pregnancy intention only at annual visits. It may be that OKQ was viewed as more of a distraction, particularly during problem focused visits, in the family medicine clinic versus the OB/GYN clinic because patients often seek care for a wide range of needs in a family medicine clinic, often nonreproductive.

Further research should focus on ways to better adapt the OKQ gold standard protocol for family medicine settings to optimize its effectiveness as a screening tool while not overburdening clinicians. Another area of future research could be the effect of OKQ on patient satisfaction given the patient's expectations of the visit agenda. In visits where the expectation is a problem-focused encounter, OKQ may be viewed as distracting from the patient's concerns, whereas in well-visits, it may be viewed as part of a comprehensive care approach.

One limitation of this study is that it focuses on the implementation of OKQ at only two practices, both associated with the same health system. Data gathered may not be reflective of the facilitators and barriers of OKQ implementation that would emerge in other types of sites. However, by using a theory-based framework for implementation assessment, we sought to make these findings more generalizable. Check-in time, room turnover, or visit length was not quantified, thus objective evaluation of actual workflow impact is not possible; however, data from the surveys and interviews indicate that clinicians and staff did not think OKQ had a major impact on clinic workflow overall.

## Conclusions

The perceived benefits held by staff and clinicians of OKQ served as observable evidence that the tool not only aligned with their goals to provide quality reproductive health care to patients but also it would help them accomplish it, providing more motivation to ask and address OKQ consistently. Nonetheless, agreement with the concept of pregnancy intention screening does not necessarily come with support of the OKQ gold standard approach of screening at every visit, and the effectiveness of the tool may depend on adapting workflows to facilitate its use without always involving providers' time.

In conclusion, results from the study indicate the importance of intervention characteristics and inner setting characteristics in the successful implementation of OKQ. When implementing a novel intervention to address reproductive health in primary care and general OB/GYN, it is important to involve stakeholders and gain their investment in the intervention's success, particularly the perceived alignment of an intervention's goals and the goals of clinicians and staff. Characteristics of the clinical setting, such as time constraints and competing patient and provider priorities, also contribute to the perceived feasibility and can influence initial attitudes toward an intervention.

## Abbreviations Used

ACOG: American College of Obstetrics and Gynecology
CDC: Centers for Disease Control and Prevention
CFIR: Consolidated Framework for Implementation Research
OB/GYN: obstetrics and gynecology
OKQ: One Key Question^®^
